# Human Bile Contains Cholangiocyte Organoid-Initiating Cells Which Expand as Functional Cholangiocytes in Non-canonical Wnt Stimulating Conditions

**DOI:** 10.3389/fcell.2020.630492

**Published:** 2021-02-09

**Authors:** Floris J. M. Roos, Monique M. A. Verstegen, Laura Muñoz Albarinos, Henk P. Roest, Jan-Werner Poley, Geert W. M. Tetteroo, Jan N. M. IJzermans, Luc J. W. van der Laan

**Affiliations:** ^1^Department of Surgery, Erasmus Medical Center, University Medical Center Rotterdam, Rotterdam, Netherlands; ^2^Department of Gastroenterology and Hepatology, Erasmus Medical Center, University Medical Center Rotterdam, Rotterdam, Netherlands; ^3^Department of Surgery, IJsselland Hospital, Capelle aan den IJssel, Netherlands

**Keywords:** cholangiocytes, bile, organoids, minimal invasive, human, non-canonical Wnt

## Abstract

Diseases of the bile duct (cholangiopathies) remain a common indication for liver transplantation, while little progress has been made over the last decade in understanding the underlying pathophysiology. This is largely due to lack of proper *in vitro* model systems to study cholangiopathies. Recently, a culture method has been developed that allows for expansion of human bile duct epithelial cells grown as extrahepatic cholangiocyte organoids (ncECOs) in non-canonical Wnt-stimulating conditions. These ncECOs closely resemble cholangiocytes in culture and have shown to efficiently repopulate collagen scaffolds that could act as functional biliary tissue in mice. Thus far, initiation of ncECOs required tissue samples, thereby limiting broad patient-specific applications. Here, we report that bile fluid, which can be less invasively obtained and with low risk for the patients, is an alternative source for culturing ncECOs. Further characterization showed that bile-derived cholangiocyte organoids (ncBCOs) are highly similar to ncECOs obtained from bile duct tissue biopsies. Compared to the previously reported bile-cholangiocyte organoids cultured in canonical Wnt-stimulation conditions, ncBCOs have superior function of cholangiocyte ion channels and are able to respond to secretin and somatostatin. In conclusion, bile is a new, less invasive, source for patient-derived cholangiocyte organoids and makes their regenerative medicine applications more safe and feasible.

## Introduction

Cholangiopathies are associated with significant morbidity and mortality (Murray and Carithers, 2005; Levitsky, 2011). Insight in the underlying pathophysiology and treatment options is incomplete, mostly due to the lack of good model systems to culture and expand primary human cholangiocytes (Sampaziotis et al., 2015). Recently, Sampaziotis et al. (2017) described a method to expand primary human cholangiocytes from extrahepatic bile duct biopsies and culture them as three-dimensional (3D) organoids. These so-called extrahepatic cholangiocyte organoids (ncECOs) retained most biliary characteristics in culture and were successfully used to bioengineer artificial ducts that, after transplantation in mice, act as functional bile ducts. The culture of ncECOs is driven by non-canonical Wnt-signaling (ncECOs) stimulated by R-spondin in combination with Dickkopf-related protein-1 (DKK-1). Also *in vivo*, non-canonical Wnt-stimulation is important for cholangiocyte homeostasis and proliferation responses to bile duct injury (Cui et al., 2011; Strazzabosco and Fabris, 2012). Although highly effective, to grow these organoids either a tissue biopsy from the gallbladder or a brush via an endoscopic retrograde cholangiopancreatography (ERCP) is needed (Sampaziotis et al., 2017; Tysoe et al., 2019). These options are potentially harmful to patients (Seeff et al., 2010) or are procedures not regularly performed. This limits broad patient-specific disease modeling and regenerative medicine applications. To avoid the need for tissue-derived samples, we explored the use of human bile as an alternative, less invasive, source for patient-derived ncECOs. A recent study published by Soroka et al. (2019) showed feasibility of culturing cholangiocyte organoids from bile, obtained by ERCP. The expansion of these bile-derived organoids were driven by canonical Wnt-signaling originally described for the growing intrahepatic cholangiocyte organoids (cICOs) (Huch et al., 2015). This canonical Wnt-signaling is stimulated by R-spondin, providing a cholangiocyte with a more stem cell-like phenotype compared to *in vivo* cholangiocytes (Sato et al., 2009, 2011; Barker et al., 2010; Soroka et al., 2019; Rimland et al., 2020). Moreover, these canonical Wnt stimulation culture conditions will likely result in a different cell phenotype than observed under non-canonical Wnt conditions (Sampaziotis et al., 2017). Since bile duct tissue-derived ECOs cultured in canonical-Wnt conditions (cECOs) do upregulate Wnt-target genes, while ncECOs do not (Sampaziotis et al., 2017; Verstegen et al., 2020). Currently, it is unknown whether under non-canonical Wnt-stimulated conditions cholangiocyte organoids can be expanded from bile and retain a more cholangiocyte-like phenotype. Therefore, the aim of this study is to initiate and expand non-canonical Wnt driven cholangiocyte organoids from bile (ncBCOs) and to compare these to canonical-Wnt driven bile cholangiocyte organoids (cBCOs) from the same bile samples and to ncECOs from (paired) bile duct tissue.

## Materials and Methods

### Bile and Tissue Collection

Fresh bile (1 ml) was collected from patients receiving an ERCP (*n* = 8) for their regular treatment (complete list of patients see Supplementary Table 1). In addition, bile (3 ml) was collected *ex vivo* from gallbladders after surgical removal (*n* = 8). Bile from gallbladders was collected from donor livers allocated for liver transplantation (*n* = 8). Additionally, tissue samples were collected from gall bladders (*n* = 5), either collected after cholecystectomy (*n* = 1) at the IJsselland Hospital, Capelle aan de IJssel, Netherlands, or were along with liver biopsies (*n* = 3) or extrahepatic bile duct (EHBD, *n* = 4) biopsies, collected from gall bladders from donor livers allocated for liver transplantation (*n* = 4). All bile samples were stored immediately on ice and were processed as soon as possible after collection. All patients or their next of kin consented with the use of their bile or tissue for research purposes by signing an informed consent, and the use of this material was approved by the Medical Ethical Committee of the Erasmus MC, Rotterdam (MEC-2014-060, MEC-2016-743, and MEC 2018-1174).

### Generation and Culture of Canonical and Non-canonical Wnt Stimulated Cholangiocyte Organoids From Bile and Tissue

For the initiation (of culture) of canonical Wnt stimulated bile or intrahepatic or extrahepatic bile duct tissue cholangiocyte organoids (cBCOs, cICOs, and cECOs, respectively), and non-canonical Wnt stimulated extrahepatic bile duct tissue (ncECOs), we used protocols similar to the ones previously published (Huch et al., 2015; Sampaziotis et al., 2017; Soroka et al., 2019; Rimland et al., 2020; Verstegen et al., 2020). For detailed methodology, please refer to Supplementary Material and Methods. Human ncBCOs (*n* = 16) were cultured from 1 ml (ERCP) or 3 ml of bile (gallbladder), depending on the source. Bile was centrifuged at 453 *g* for 5 min at 4°C, supernatant was removed, and the cell pellet was washed twice with excess cold William’s-E medium (WE). Finally, the pellet was suspended in WE and filtered through a 70-μm cell strainer to remove debris. Afterward, cells were plated out in a 25-μl droplets of basement membrane extract (BME, Cultrex) and culture medium (WE with supplements) was added according to the standard ncECO protocol (Sampaziotis et al., 2017; Tysoe et al., 2019). Medium was changed twice a week, and cultures were split in a 1:2 to 1:10 ratio depending on the number and size of organoids grown. Cultures were routinely checked for mycoplasma contamination, which came back negative. All experiments with ncECOs and ncBCOs were performed with passage five or higher, unless stated otherwise. For a complete overview of the nomenclature and culture conditions for cholangiocyte organoids and patient characteristics, see Supplementary Tables 1, 3.

### Flow-Cytometry of Bile-Derived Cells and ncBCOs

Fresh human bile was obtained from patients immediately after collection via ERCP (*n* = 3) and stored at 4°C during transportation. Bile was transferred to a 15 mL Falcon tube and centrifuged for 5 min at 4°C, supernatant was removed, and the cell pellet was washed twice with WE. ncBCOs were made single cell by incubation with Trypsin-EDTA (TE) for 25 to 40 min, at 37°C. Cells were washed in WE and put through a cell 70 μm cell strainer. Samples were blocked in 1% bovine serum albumin (BSA)-Phosphate-buffered saline (PBS) for 15 min. TROP2 antibody (Invitrogen; rabbit monoclonal conjugated to Alexa Fluor-488, clone MR54, used 1:100) was added (30 min, on ice), and cells were subsequently measured on a Canto flow cytometer (BD Biosciences).

### RNA Extraction, cDNA Synthesis, and RT-qPCR

RNA was harvested by the addition of 700 μl of QIAzol lysis reagent (Qiagen) per two 25-μl domes of ncECO (*n* = 6), ncBCO (*n* = 6), and cBCOs (*n* = 6). RNA extraction and subsequent cDNA synthesis was performed as previously published (Roest et al., 2019). In short, RNA was isolated using an miRNeasy kit (Qiagen) according to the manufacturer’s protocol. RNA concentration was measured using a NANOdrop 2,000 (Thermo Fisher). cDNA (500 ng) was prepared using 5× PrimeScript RT Master Mix in a 2,720 thermal cycler (Applied Biosystems). RT-qPCR was performed with the primer sets provided in Supplementary Data Table 2. All RT-qPCR data are presented as mean with a 95% confidence interval. RT-qPCR values are relative to the housekeeping gene Hypoxanthine-guanine-fosforibosyl-transferase (*HPRT*) or Glyceraldehyde 3-phosphate dehydrogenase (*GAPDH*) and for visual interpretation multiplied by 10^5^ or 10^6^.

### Immunohistochemistry

Immunohistochemistry (IHC) was performed according to standard procedures as previously described for cICOs; liver biopsies obtained from donors allocated for transplantation were taken along as control (Huch et al., 2015). In short, formalin-fixed, paraffin-embedded bile duct biopsies were sectioned (4 μm thick) and processed according to standard procedures. Antigen retrieval was performed in citrate buffer (pH 6.0) for 10 min in sub boiling temperatures and non-specific reactions were blocked by incubation with 10% goat serum in a 1% BSA-PBS solution. The sections were exposed to primary antibodies overnight at 4°C Cytokeratin (KRT)19 and KRT7 antibodies (both Dako were used in 1:100 dilution in 1% BSA-PBS). The antibody for Cystic Fibrosis Transmembrane Conductance Regulator (CFTR, EMD Millipore Corp.) was used in a 1:200 dilution of 1% BSA-PBS. Sections were subsequently incubated with Envision + system horseradish peroxidase anti-mouse secondary antibody (Dako) at room temperature for 60 min, before staining with 3′-diaminobenzidine (DAB). Nuclei were stained by hematoxylin. Analysis was done with a Carl Zeiss Axioskop 20 microscope, and images were taken with a Nikon Digital Side DS-5M camera.

### Gene-Array Selection-Based qRT-PCR

**FIGURE 1:**
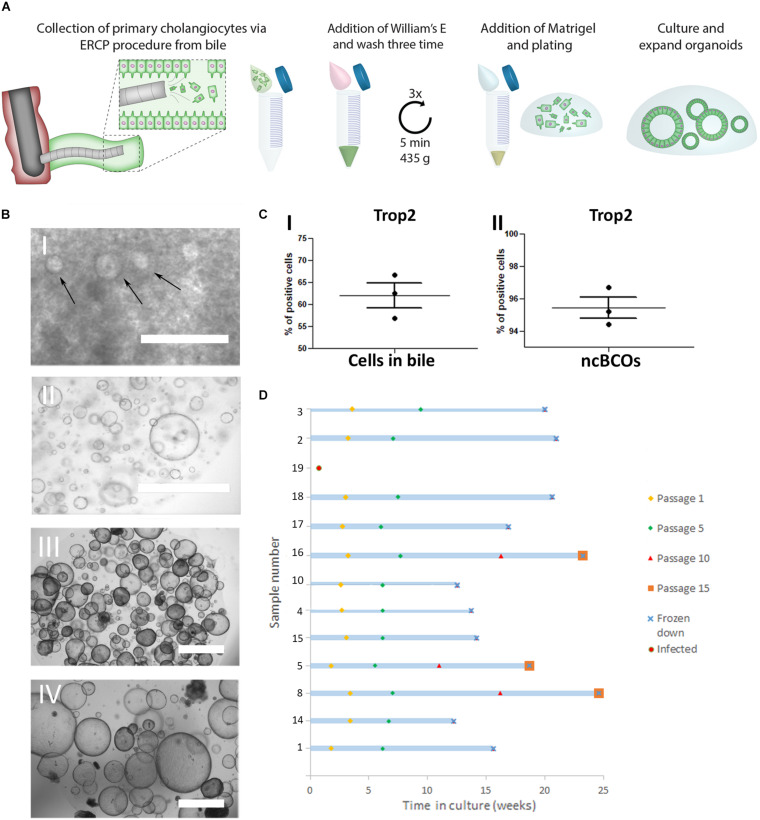
Creation and culturing of bile cholangiocyte organoids in non-canonical Wnt stimulating conditions (ncBCOs). **(A)** Schematic representation of initiation of ncBCOs from endoscopic retrograde cholangiopancreatography (ERCP) collected bile. First bile is collected via an ERCP-procedure (1 ml) Organoid-initiating cells (viable cholangiocytes) are present in bile and bile should be quickly transferred on ice for processing. Next the bile was diluted and washed a total of three times with William’s-E medium, to remove all bile from the cells. If a lot of debris is present, additional cells could be passed through a 70 μm cell strained. Subsequently, a hydrogel (Matrigel, Corning or base membrane extract, Cultrex) is added and the cells are plated out in 25 μl of hydrogel droplets and cholangiocytes are expended as organoids. **(B) (i)** A representative image of ncBCOs at passage 0 (P0) after 5 days in culture. **(ii)** P5 ncBCOs showing mostly cyst-like organoids. **(iii)** A representative of an extrahepatic cholangiocyte organoid in non-canonical Wnt stimulating conditions (ncECO) P3. **(iv)** ncECO passage P5. All scale bar indicates 1,000 μm. **(C) (i)** Trop2 flow cytometry of all cells counted as an event in bile, indicating that Trop2^pos^ cells are dominantly present in bile, in **(ii)** the mean percentage of Trop2^pos^cells in ncBCOs are displayed. **(D)** Number of passages of the 13 ncBCOs cultured before cryopreservation and storage.

Twenty genes were selected from the online published Microarray gene expression data corresponding to the heat map (Figure 1D) published in Sampaziotis et al. (2017). Out of the gene-expression heatmap, 10 genes were selected, which have a similar expression between primary human cholangiocytes and ncECOs, five genes were selected, which have higher expression in ncECOs compared with primary human cholangiocytes and five genes, which have lower expression in ncECOs compared with primary human cholangiocytes. Additionally, 15 genes known to be Wnt-target/stem cell-related or hepatocyte and cholangiocyte specific, were selected (Huch et al., 2015), and their expression was assessed by RT-qPCR. The selected genes and correlating primer sets are listed in Supplementary Data Table 2. The details on the ncECOs and ncBCOs used are described in Supplementary Data Table 1. A Z-stack heat-map was created by unsupervised hierarchical clustering in R (version 3.5.1, R Core Team) supplemented with package Gplots (version 3.0.1).

### Swelling of Cholangiocyte Organoids

To assess functionality of secretin and somatostatin, a slight modification of the Forskolin-Induced Swelling (FIS) assay as developed by Dekkers et al. (2013) was applied. For this, ncECOs and ncBCOs organoids were incubated for 30 min with 3 μM calcein-green (Invitrogen), stimulated with secretin (10 μM) or secretin and somatostatin (100 μM) and analyzed by confocal live cell microscopy at 37°C for 120 min (LSM710, Zeiss). The total or single organoid area (XY plane) increase relative to *t* = 0 of secretin treatment was quantified using velocity imaging software (Improvision) and compared to non-stimulated controls. Cell debris and unviable structures were manually excluded from image analysis.

### Gamma-Glutamyltransferase Assay

Ten microliters of supernatant of cholangiocyte organoids from bile (ncBCOs, *n* = 3) and from tissue (ncECOs, *n* = 3) were collected. Next, Gamma-glutamyltransferase (GGT) activity colorimetric assay kit (MAK089; Sigma-Aldrich) was used according to the manufacturer’s protocol.

### Rhodamine 123 Transport Assay

Functionality of the multi drug resistance-1 (MDR-1) transporter was performed according standard protocol using a commercially available Rhodamine 123 assay (Sigma Aldrich) (Sampaziotis et al., 2017). Rhodamine 123 transport was determined by incubating the cultures with Rhodamine 123 (100 μM, Sigma Aldrich) for 5°min at 37°C. Subsequently, specificity of the MDR-1 transporter was determined by blocking the transporter with Verapamil (10°μM, Sigma Aldrich) for 30°min at 37°C prior to Rhodamine 123 incubation (100°μM, Sigma Aldrich). Confocal analysis was performed to determine MDR-1 activity. Images were acquired with a Leica SP5 confocal microscope (LEICA) equipped with a 488-nm laser.

### Ussing Chamber Assay

Extrahepatic cholangiocyte organoids, ncBCOs, cICOs, and cBCOs (all *n* = 3) were collected from 10–25 μl domes. Organoids were collected in WE, centrifuged (453 g, 5 min, 4°C) and the supernatant was removed. Organoids were mechanically broken by pipetting up and down and were spun down again. A single cell suspension was made through incubation of the organoids in 1 ml TE for 25 to 40 min, at 37°C. Cells were washed in WE and put through a cell 70 μm cell strainer. Approximately, 3 × 10^5^ cells were resuspended in 200 μm WE-medium with supplements (Sampaziotis et al., 2017) and seeded on Transwell inserts (24-well plate 6.5 mm, Corning). Medium was changed twice per week. To check confluency, electrophysiological analysis (TEER) was performed after 4 days. The confluent Transwells were placed in an Ussing chamber set up to analyze functional cholangiocyte-specific transporter channels (CFTR and Ca^2+^ dependent Cl^–^ channel) using Acquire and Analyze Software 2.3 (Physiologic Instruments, San Diego, CA, United States). For detailed methodology of the conditions, please see Supplementary Material and Methods.

### RNA Isolation for Microarray

Total RNA was isolated from cECOs using the miRNEAsy mini kit (Qiagen, Hilden, Germany) according to the manufacturer’s protocol and eluted in 30 μl of RNAse-free water. RNA concentration and integrity were determined using a Nanodrop 2000 (Thermo Fisher Scientific, Waltham MA, United States) and a Bioanalyzer 2,100 (Agilent Technologies, Santa Clara CA, United States), respectively. A total of 300 ng RNA was reversed transcribed, amplified, and biotin-labeled using the Illumina TotalPrep RNA Amplification Kit (Ambion-Life Technologies, Carlsbad CA, United States) according to the manufacturer’s guidelines. HumanHT-12 v4 Expression BeadChips (Illumina, San Diego CA, United States) were overnight hybridized with 750 ng cRNA, washed, stained, and scanned on an iScan and analyzed using GenomeStudio V2011.1 software (both from Illumina, Inc.).

### Microarray Analysis

Microarray analysis was based upon the publically available data of ncECOs from ArrayExpress E-MTAB-4591 [Sampaziotis et al. (2017), Nature Medine 2017, *n* = 3], cICOs (*n* = 3) from the ArrayExpress E-MTAB-9044 (Roos et al. unpublished), and the novel data generated for cECOs (deposited at Array Express, E-MTAB-9807). Bead types missing in one or more arrays were excluded, and the resulting non-normalized raw probe data set was combined with the open datasets. The file, describing for each probe AVG_Signal and Detection Pval, was loaded into R using the limma package (Ritchie et al., 2015). limma powers differential expression analyses for RNA-sequencing and microarray studies. Nucleic Acids Research 43, e47). Probes that were present at least once (Detection Pval <0.01) were considered as being expressed (21,929 probes) and used for further analysis. Following filtering, the data were background subtracted, normalized, and log2 transformed using the VSN package (Huber W, von Heydebreck A, Sueltmann H, Poustka A, Vingron M (2002). “Variance Stabilization Applied to Microarray Data Calibration and to the Quantification of Differential Expression.” Bioinformatics, 18 Suppl. 1, S96-S104). The heatmap was conducted in R using heatmap.2 functions.

### Statistical Analysis

All analyses were conducted using SPSS software (statistical Product and Service solutions, version 22, SSPS Inc., Chicago, IL, United States), and graphs were performed using GraphPad Prism 7.0 (GraphPad Software Inc., United States). Continuous variables were tested using an independent T-test or Mann–Whitney-*U* test and presented with normal distribution as means with standard error of the mean and if not normally distributed, they are presented as range. Gene array unsupervised hierarchical clustering data was statistically analyzed using Pearson’s correlation was calculated between the eight samples (reference ncBCO 10). In all tests, a *P*-value of <0.05 was considered significant.

## Results

### Cholangiocyte Organoids in Non-canonical Wnt Stimulating Conditions Can Successfully Be Expanded From Human Bile

In Figure 1A, a schematic overview of the culture protocol for bile cholangiocyte organoids (ncBCOs) from bile is displayed. As shown in Figure 1B, ncBCOs could be successfully cultured from bile obtained from multiple organ donors or patients with different underlying liver or biliary diseases (*n* = 15). Donor, patient, and culture characteristics are shown in Supplementary Data Table 1. NcBCOs could be initiated from both ERCP- and gallbladder bile with a high success rate (15 from 16, 94%). Morphologically ncECOs and ncBCOs looked similar under bright-field microscopy (Figure 1B). Recent evidence showed that primary human cholangiocytes are Trop2 positive (Aizarani et al., 2019). Thus, to investigate if primary cholangiocytes are present in bile we looked at Trop2^pos^ cells. As shown in Figure 1C, the Trop2^pos^ cell population was the highly dominated population within the bile samples (mean percentage 62.20 ± 2.69). To confirm that ncBCOs resemble their Trop2^pos^ organoid-initiating population *in vitro*, we performed flow cytometry analysis on ncBCOs. As indicated by Figure 1C, almost all cells found within organoids are indeed Trop2^pos^ (mean percentage 95.43 ± 0.67) highlighting their resemblances to primary cholangiocytes. Similar to ncECOs, ncBCOs rapidly expanded and could be passaged approximately one time a week in a 1:3 ratio. ncBCOs could be passaged for at least 15 passages (>3 months) (Figure 1D).

### ncBCOs and ncECOs Have Similar Gene- and Protein-Expression Profiles

**FIGURE 2:**
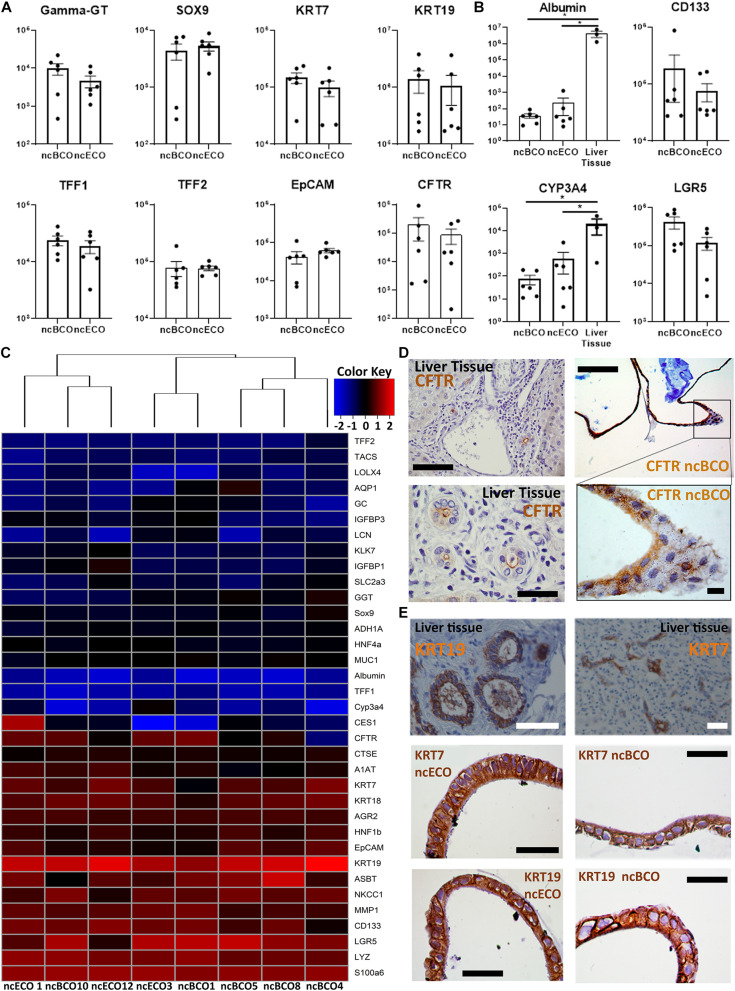
Gene and protein expression between bile-derived cholangiocyte organoids (ncBCOs) and extrahepatic cholangiocyte organoids (ncECOs) is similar. **(A)** Gene expression profiles of selected genes between ncECOs (*n* = 6) and ncBCOs (*n* = 6) for eight mature cholangiocyte-related genes relative to the reference gene hypoxanthine-guanine-fosforibosyl-transferase (*HPRT*) (for characteristics of organoid lines or gene and primer details see Supplementary Data Tables 1, 2). Error bars are presented as standard error of the mean (SEM). **(B)** Gene expression profiles of selected hepatocyte and stemness/Wnt-target-genes between ncECOs (*n* = 6), ncBCOs (*n* = 6), and liver-biopsies (*n* = 3) relative to the reference gene *HPRT*, indicating that ncECOs and ncBCOs have very low expression of hepatocyte-related genes: Albumin and Cyp3A4, and they have similar expression of stemness/Wnt-target-related genes. Error bars are presented as SEM. **(C)** Gene-expression profiles of ncBCOs and ncECOs are highly similar for these 35 selected genes. The included genes were selected based on published results by Sampaziotis et al. (2017)Figure 1D along with additional Wnt-target, cholangiocyte and hepatocyte related genes (Huch et al., 2015) and were quantified by qRT-PCR (see Methods and Supplementary Data Table 2). Euclidean hierarchical clustering did not show a significant segregation of gene-expression between ncECOs and ncBCOs. **(D)** Immunohistochemistry (IHC) staining of cystic fibrosis transmembrane conductance regulator (CFTR) in ncBCOs showing polarization of our organoids and luminal CFTR expression, similar to liver biopsies, top row scale bars indicate 200 μm, bottom scale bars indicate 50 μm. **(E)** Immunohistochemistry of ncBCOs and ncECOs showing comparable staining of cytokeratin, (KRT)19, and KRT7. Moreover, it reveals a columnar-like cell, resembling primary cholangiocytes as shown on liver tissue coupes. All scale bars in IHC pictures of ncECOs and ncBCOs represent 25 μm, and all scale bars from the liver biopsy pictures represent 100 μm.*Statistical significant difference (*P* < 0.05).

To determine the phenotype of ncBCOs, gene-expression of a selected number of cholangiocyte-specific genes was assessed and compared to ncECOs using qRT-PCR (Sampaziotis et al., 2017). Organoid lines included for qRT-PCR analysis were almost all (four out of six) paired from the same donor to overcome donor variances. Additionally, samples were from patients with a similar disease (ncBCO8 and ncECO11), or with a relatively healthy bile duct (ncBCO10 and ncECO12). Details of the origin of the organoids are shown in Supplementary Table 1). Expression of cholangiocyte markers KRT19, KRT7, and hepatocyte nuclear factor-1beta (HNF1β), CFTR, Trefoil Factor 1 (TFF1), Trefoil Factor 2 (TFF2), and biliary progenitor marker sex-determining region Y (Sox)9 was assessed. As shown in Figure 2A, these cholangiocyte markers were highly expressed in ncECOs and ncBCOs and no significant differences in expression was observed. As a control, the expression of hepatocyte markers albumin (ALB) and CYP3A4 compared to liver-tissue biopsies, as well as the Wnt-target gene leucine-rich repeat-containing G-protein coupled receptor 5 (LGR5) and the stem cell marker prominin-1, also known as CD133, was determined (Figure 2B). Compared to liver biopsies that mostly consists of hepatocytes, the expression of the hepatocyte markers albumin and Cyp3A4 was 17,500–114,970 times and 32–260 times lower in ncECOs and ncBCOs, respectively (Figure 2B), highlighting the resemblance of ncECOs and ncBCOs with cholangiocytes. Furthermore, no differences in gene-expression for LGR5 and CD133 could be determined between ncECOs and ncBCOs. To further confirm that ncBCOs have a similar gene expression profile as ncECOs, the expression of 35 genes (for gene selection see Material and Methods) was determined in ncBCOs and ncECOs using cluster analyses. Pearson correlation coefficient showed no major differences between the expression of these genes in both organoid types (Figure 2C). To confirm that the organoids are polarized, a CFTR-staining was performed, as shown in Figure 2D. CFTR is predominantly expressed on the luminal side of our ncBCOs, resembling the *in vivo* situation in the liver (Figure 2D). Furthermore, IHC staining of KRT7 and KRT19 confirmed expression of both cholangiocyte markers in the cytoplasm of ncBCO and ncECOs. Moreover, histology revealed a typical columnar-like epithelium in the organoids, with the nucleus located basolateral, similar to primary cholangiocytes in tissue biopsies (Figure 2E) (Boyer, 2013).

### Both ncBCOs and ncECOs Have Cholangiocyte Functionality *in vitro*

**FIGURE 3:**
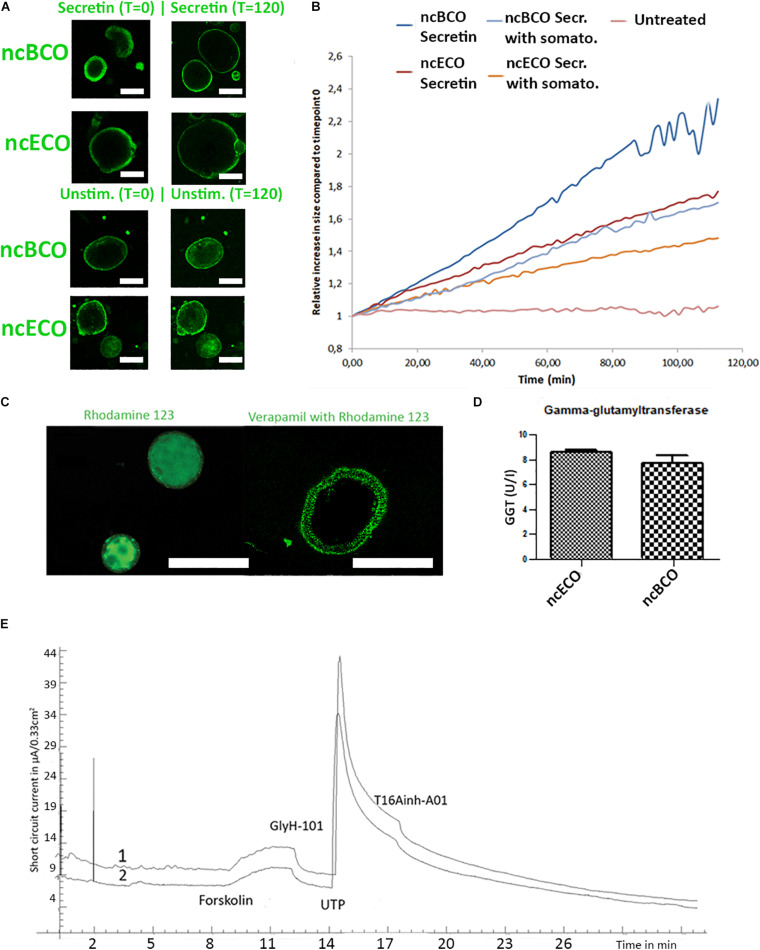
ncBCOs and ncECOs both have functional cholangiocyte activity. **(A)** Representative images of selected ncECOs and ncBCOs at time is 0 min, and time is 120 min of measurements in the secretin and unstimulated (unstimulated) groups. **(B)** Quantification of organoid swelling in ncECOs and ncBCOs in different groups, scale bars indicate 50 μm. **(C)** ncBCOs show clear multi drug resistance-1 (MDR-1) activity as Rhodamine 123 was actively transported out of the cells into the lumen of the organoid. Specificity was confirmed by inhibition with Verapamil. **(D)** Gamma-glutamyltransferase (GGT) activity (U/I) as measured by ELISA in both ncECOs and ncBCOs supernatants (*n* = 3), scale bars indicate 100 μm. **(E)** Representative ion-channel functionality of 2D-grown ncBCOs (line 1) and ncECOs (line 2) in an Ussing chamber. Stimulation with cAMP-activator, forskolin, resulted in an increase in short circuit current, demonstrating CFTR-mediated activity (Cystic Fibrosis Transmembrane conductance Regulator). This was completely blocked by CFTR-inhibitor, GlyH-101. Also, calcium-depended chloride excretion ion channel activity, specifically stimulated by UTP and inhibited by T16Ainh-A01, was identical between ncBCOs and ncECOs.

Cholangiocytes influence bile quality by secretion of ions via either CaCl and Anoctamin-1 (ANO1) transporter channels or via an increase of cAMP, which regulates CFTR (Boyer, 2013). Basolateral secretin and somatostatin receptors regulate intracellular cAMP concentrations (Boyer, 2013). As displayed in Figures 3A,B, ncBCOs responded to stimulation of secretin similar to the *in vivo* situation by showing an increase (2.21 ± 0.48 fold change) in diameter (*t* = 120) compared to *t* = 0 min. The ncECOs responded similarly (mean fold change 1.74 ± 0.22). Addition of somatostatin reduced the induced swelling (1.69 ± 0.187 increase in ncBCOs and 1.41 ± 0.14 for ncECOs), confirming functional secretin and somatostatin receptors in ncBCOs.

The capability to export drugs was confirmed in ncBCOs by imaging effective transport of Rhodamine 123 by the multidrug resistance protein-1 (MDR1) (Sampaziotis et al., 2017). The complete abrogation of fluorophore transport to the lumen of the organoids by Verapamil, confirmed MDR-1 dependency (Figure 3C). Both findings are in line with the previously published results for ncECOs (Sampaziotis et al., 2017).

Previous work by Sampaziotis et al. (2017) showed that activity of GGT in ncECOs is similar to primary cholangiocytes. To investigate if the GGT activity of ncBCOs is in line with ncECOs, we cultured both ncECOs and ncBCOs and analyzed the culture supernatant for GGT-activity. As shown in Figure 3D we demonstrate that GGT excretion is similar between ncBCOs and ncECOs (7.77 ± 1.10 vs. 8.59 ± 0.34, *p* = 0.63) as measured by ELISA. Since the activity of ncECOs and primary cholangiocytes is also similar, it seems plausible that ncBCOs resemble GGT activity similar to that of primary cholangiocytes.

Organoids are normally grown in a 3D setting, making it difficult to access the cells’ luminal side. In cholangiocytes, both the ANO1 and CFTR channel are located at the luminal side. To overcome the hurdle of accessing this side, both ncBCOs and ncECOs were grown as 2D monolayers and ion-channel functionality was addressed in an Ussing chamber. As shown in Figure 3E, ncECOs and ncBCOs both responded to activation with forskolin (cAMP-activator), which was completely inhibited by addition of GlyH-101, a CFTR-inhibitor, to the luminal side. Additionally, stimulation by UTP (luminal) and inhibition (by T16Ainh-A01, luminal) demonstrated the presence of functional ANO1 in both organoid types. Combined, this demonstrates that ncBCOs and ncECOs are very similar type organoids that both represent cholangiocyte-like characteristics.

### ncBCOs Retain Secretin Receptor Responsiveness and Increased Ion-Channel Activity *in vitro* Compared to cBCOs

**FIGURE 4:**
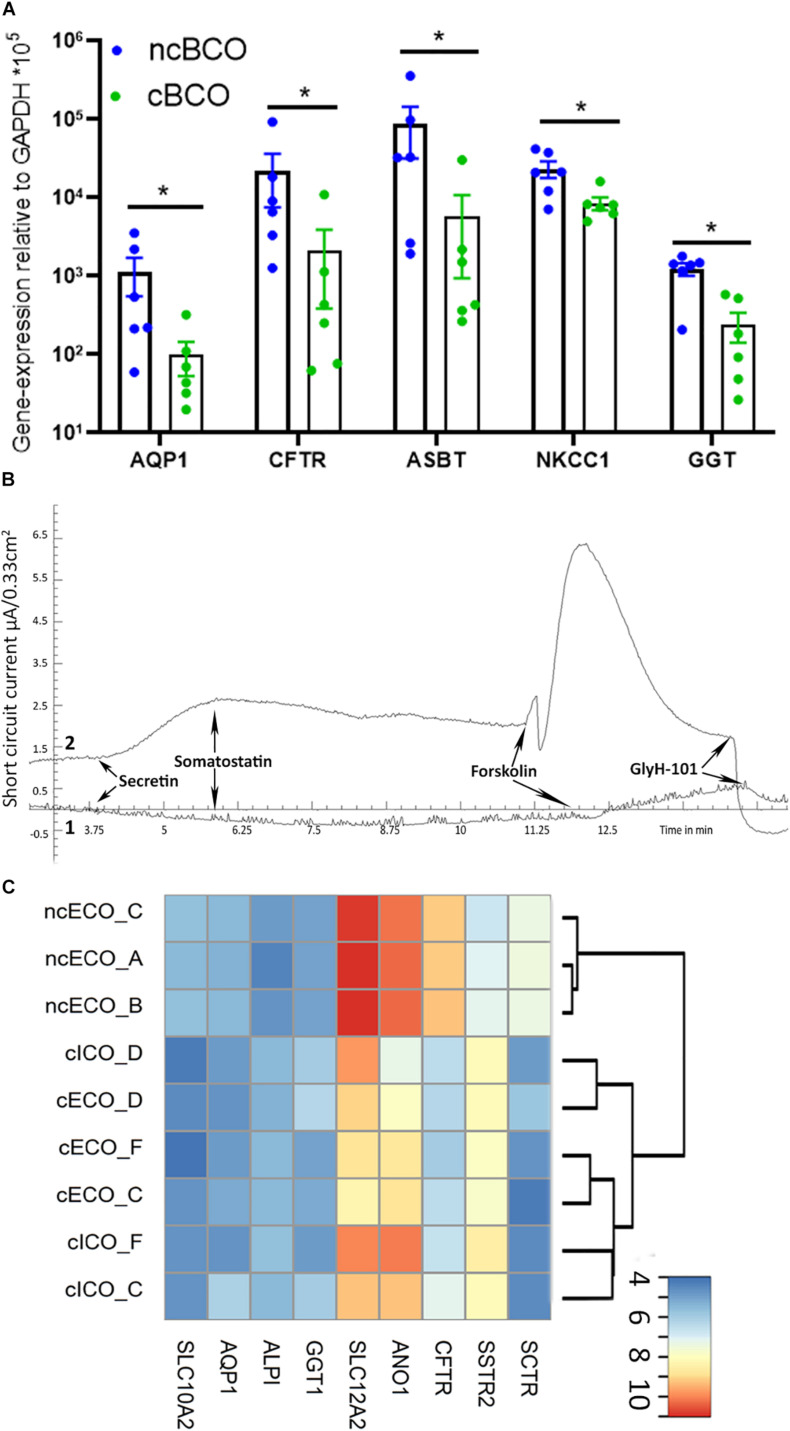
ncBCOs retain secretin receptor responsiveness and increased ion channel activity *in vitro* compared to cBCOs. **(A)** qRT-PCR of ncBCOs and cBCOs (both, *n* = 6), for cholangiocyte specific channels and transporters. Showing a significant upregulation (*p* < 0.05, as indicated by *) for AQP1, CFTR, ASBT, NKCC-1, and GGT in ncBCOs compared to cBCOs. Error bars are displayed as SEM. **(B)** Representative ion-channel functionality of 2D-grown bile-cholangiocyte organoids in non-canonical Wnt-stimulated conditions (ncBCOs, line 2) and bile cholangiocyte organoids in canonical-Wnt-stimulated organoids (cBCOs, line 1) in an Ussing chamber, stimulation with cAMP-activator (forskolin), resulted in an increase in short circuit current; however, secretin stimulation (to the basolateral side) only gave a response in the ncBCOs. In similar fashion, somatostatin (basolateral addition) only give a response in ncBCOs and not in cBCOs, while CFTR inhibition via GlyH-101 (luminal addition), resulted in an inhibition of the channel in both organoid-types, indicating the presence of functional CFTR channels in both organoids, but only somatostatin and secretin receptors are functional in ncECOs. Moreover, it seems that CFTR-function is higher in organoids in non-canonical Wnt stimulating conditions compared to organoids in canonical-Wnt-stimulating conditions. **(C)** Heatmap and clustering based expression of cholangiocyte-related gene expression as analyzed by gene array for functional enzymes (ALPI and GGT1), channels (SLC12A2—also known as NKCC1-, SLC10A2—also known as ASBT-, AQP1, ANO1, and CFTR) and receptors (SCTR and SSTR) between cICOs (*n* = 3), cECOs (*n* = 3), and ncECO (*n* = 3). Color key represents the log2 transformed signal intensities after variance stabilizing normalization.

Soroka et al. (2019) created bile-cholangiocyte organoids (cBCOs), using the established culture conditions by Huch et al. created for cICOs (Huch et al., 2015). To investigate if there are differences between ncBCOs and cBCOs, we created organoids from bile samples and cultured them using both protocols (*n* = 3). We analyzed gene-expression profiles using qRT-PCR. First, we assessed expression of the Wnt target gene LGR5 and several cholangiocyte-related genes. As indicated in Supplementary Figure 1A expression of all genes tested was similar in ncBCOs and cBCOs (Supplementary Figure 1A). To investigate potential donor-variations between samples, we also compared the expression profiles between cECOs and ncECOs for the same cholangiocyte and Wnt-target genes by micro-array. As shown in Supplementary Figure 1B, the expression profiles of biological replicates for both culture conditions did not differ. Moreover, the overall expression profiles of cECOs and ncECOs are similar, although the differences in culture conditions seems to cause clustering of specific genes, indicating that this is an important factor driving gene-expression profiles. In line with this observation, the mean gene-expression of functional cholangiocyte channels, transporters, and enzymes NKCC1, ASBT, CFTR, GGT, and AQP1 are significantly higher in ncBCOs compared to cBCOs (Figure 4A). This was an interesting finding and to further confirm differences in functional receptors and ion channels, we performed the Ussing chamber assay using 2D grown ncBCOs and cBCOs. As a control both the tissue-derived organoids (ncECOs and cICOs) from the original publications were created (Huch et al., 2015; Sampaziotis et al., 2017). As shown in Figure 4B and Supplementary Figure 2 only organoids cultured in the non-canonical Wnt-stimulated medium (ncBCOs and ncECOs) could respond to addition of secretin and somatostatin. This indicates that functional SCTR and SSTR are only present in cholangiocyte-organoids cultured in this specific medium. In contrast, organoid cultured in the canonical-Wnt stimulated conditions (cICOs and cBCOs) did not respond to secretin or somatostatin. Moreover, it seems that although all four-organoid types could respond to forskolin and this response was specifically inhibited by addition of GlyH-101 (a specific CFTR-inhibitor), demonstrating functional CFTR-channels in all organoids, this effect was more pronounced in organoids cultured under the non-canonical Wnt-stimulated conditions. To investigate the reason for the differences in functionality of cholangiocyte organoids in different culture conditions we looked at gene-expression profiles as analyzed by gene-array of publically available and novel generated data of cICOs, cECOs, and ncECOs. All cICOs and cECOs were matched for donors (*n* = 3). As shown in Figure 4C, the gene expression of almost all mature cholangiocyte-related enzymes, channels, and receptors (Dekkers et al., 2013) was higher in non-canonical Wnt-related conditions, similar to the results of the qRT-PCR analysis (Figure 4A). This suggests that lack of functional SCTR is the result of low or absent SCTR gene expression in organoids under canonical-Wnt conditions. Interestingly, cICOs and cECOs cluster together, indicating that culture conditions overshadows differences related to the region of origin (e.g., from intra- or extrahepatic). To further investigate the influence of culture conditions, we changed the medium in the different organoid cultures. As shown in Supplementary Figure 3A, it seems that culture conditions for the classical cholangiocyte markers do not differ in BCOs, with the exception for the expression of HNF1β between cBCOs and ncBCOs in this series. Interestingly, although BCOs are initiated in medium containing canonical-Wnt stimulation, switching them to non-canonical Wnt stimulating medium alters the gene-expression profiles for genes related to function (Supplementary Figure 3B). This change in medium directed the switched cBCO gene-expression profile to become more closely related to bile-cholangiocyte organoids initiated in non-canonical Wnt conditions and expression of ASBT and GGT was significantly higher under non-canonical Wnt stimulated conditions. A similar effect for these genes was observed when switching ncBCOs to canonical Wnt stimulating conditions (Supplementary Figure 3B). These results suggest that cholangiocyte-organoids cultured in different conditions are very similar, but differences might be induced by the culture conditions.

## Discussion

In this study, we demonstrate that primary biliary epithelial cells (cholangiocytes) collected from bile can be cultured and expanded efficiently *in vitro* while retaining their cholangiocyte characteristics. We show that these ncBCOs, could be efficiently initiated from ERCP and gallbladder bile and could be cultured long-term. In addition, we show that ncBCOs have similar characteristics when compared to ncECOs as described by Sampaziotis et al. (2017). Similar gene-expression profiles were found in both types of organoids, and both were found to behave as cholangiocytes in functional assays. This study furthermore provides evidence that ncBCOs are distinct from the previously published cBCOs (Soroka et al., 2019). The non-canonical Wnt stimulation of ncBCOs seem to preserve the cholangiocyte-specific basolateral receptor activity *in vitro*. This is the first study demonstrating expansion of non-canonical Wnt stimulated cholangiocyte organoids from bile. Recent publications show that cholangiocyte organoids in non-canonical Wnt stimulating conditions can be cultured from intra- and extrahepatic biopsies as well as from brushes collected from the common bile duct (Sampaziotis et al., 2017; Tysoe et al., 2019). The use of biopsies and ERCP brushes as a source for biliary organoids provides promising source for *in vivo* collected cholangiocytes as well. Of note, the use of brushes is associated with more complications for the patient when compared to the collection of liquid biopsies from bile (Vandervoort et al., 1999; Korc and Sherman, 2016). Surgical procedures to obtain gallbladders or biopsies are especially risk full in patients with advanced liver cirrhosis (Currò et al., 2005; Strömberg et al., 2015), a potential target population. This is obviously an unwanted risk which limits the patient-specific applications. Moreover, bile allows for inclusion of patients with rare diseases and can model disease-progression *in vitro*, as liquid biopsies can be easily collected during routine (clinical) procedures which patients already undergo for their regular treatment at progressive time-points. Thus, ERCP-derived bile is a relatively safe source for patient-derived cholangiocyte organoids. We show that bile derived from the gallbladder is also a source of cholangiocyte organoids. We included gallbladder bile-derived organoids to study donor variations (Rimland et al., 2020; Verstegen et al., 2020), since matched collection of gallbladder bile and gallbladder-tissue from the same patient could be easily accomplished. Some patients are not eligible for ERCP and thus a percutaneous transhepatic cholangiography drainage (PTCD) is performed in these specific patients. Since we demonstrate that multiple sources of bile can be used for ncBCOs, bile collected via PTCD might represent an additional source in this specific subgroup.

Cholangiocytes organoids, either initiated from tissue or bile in both culture conditions, are most likely risen from primary cholangiocytes (Sampaziotis et al., 2017; Hu et al., 2018; Aloia et al., 2019; Planas-Paz et al., 2019). In line with previous publications, we confirmed the presence of a Trop2^pos^ population in bile, which is considered the population that resembles primary cholangiocytes (Aizarani et al., 2019). Since primary cholangiocytes are most likely the cell-of-origin for these organoid cultures, these are the culture conditions that are responsible for the difference in gene-expression and functionality *in vitro*. ncBCOs are cultured in medium with components typically stimulating the non-canonical Wnt-pathway, while cBCOs are cultured in Wnt/β-catenin stimulated conditions, usually responsible for maintaining stem cell-like characteristics in cell culture. Recent evidence indicates that non-canonical Wnt signaling is important in cholangiocyte homeostasis and proliferation (Pepe-Mooney et al., 2019; Planas-Paz et al., 2019). Both publications showed that, although Wnt ligands seem important in homeostasis of biliary epithelium *in vivo*, this was independent of the canonical Wnt-related genes AXIN2, LGR5, and β-catenin. This demonstrates that it is actually non-canonical Wnt signaling which drives ductal reprogramming. In contrast, Planas-Paz et al. (2019) discovered that LGR5-depended Wnt/β-catenin signaling is necessary for expansion of intrahepatic cholangiocytes, cultured as cICOs, *in vitro*. In line with these results several publications have reported that Wnt/β-catenin-LGR5 signaling is upregulated in canonical-Wnt driven cholangiocyte-organoid cultures, while markers for mature cholangiocytes (like CFTR) are downregulated compared to primary cholangiocytes (Aizarani et al., 2019; Pepe-Mooney et al., 2019; Planas-Paz et al., 2019). Thus, suggesting that organoids initiated under these Wnt/β-catenin stimulated culture conditions require these specific signals for growth and alter their gene-expression profile by expressing Wnt/β-catenin related target genes, although this does not seem to be essential for ductal reprogramming in *in vivo* biliary epithelium.

This *in vitro* study might validate that the use of non-canonical Wnt cell culture conditions results (for some aspects) in a more mature cholangiocyte *in vitro*, compared to Wnt/β-catenin-stimulated conditions. Here we show that ncBCO have higher expression of mature cholangiocyte-channel genes compared to cBCOs. In addition, gene expression as analyzed by gene-array shows that expression of the functional cholangiocyte-related genes such as ANO1, NKCC1, CFTR, GGT, AQP1, and the basolateral receptor SCTR is higher in non-canonical Wnt-stimulated cholangiocyte organoids when compared to Wnt/β-catenin cultured organoids from tissue. Moreover, ncBCOs (and ncECOs) have functionality of the typical cholangiocyte-receptors: secretin and somatostatin while cBCOs (and cICOs) do not. Interestingly, our analysis provides evidence that there is some plasticity in cholangiocyte organoids since we demonstrate that gene-expression profiles in BCOs change depending on the culture conditions. In line with our hypothesis, the functional-related genes ASBT and GGT are upregulated when culture medium is switched from canonical Wnt to non-canonical Wnt stimulating compounds. Also other genes (HNF1β and PROM1) are differently expressed after switching. Together, our results indicate that culture conditions most probably drive the expression of specific genes in cholangiocyte organoids and that cholangiocytes might be more mature under non-canonical Wnt stimulating conditions. It is important to note that ncECO were shown capable of forming functional bile duct tissue *in vivo* after repopulating collagen scaffolds and transplantation in mice (Sampaziotis et al., 2017). The strength of using bile as a minimally invasive source of organoid-initiation cells for culturing cholangiocyte-organoids, is that it allows for patient-specific organoid cultures and thereby avoids immunological responses after transplantation.

In conclusion, our studies confirm and extend the studies previously reported on ncECOs (Sampaziotis et al., 2017), demonstrating that these cholangiocyte organoids can efficiently and reproducibly be initiated from bile collected from patients with a broad spectrum of underlying biliary diseases. Given that ncECOs and ncBCOs are highly similar, an important amenity of ncBCOs is that they are reproducibly initiated from bile, which is collected relatively easily and in a less invasive manner opposed to surgically rendered tissue biopsies. Moreover, they seem to represent a more mature cholangiocyte compared to the previously published cBCO (Soroka et al., 2019). With this, the expansion and use of cholangiocyte-organoids that are acquired with low complication hazard from patients becomes feasible for personalized disease modeling and regenerative medicine.

## Data Availability Statement

The datasets presented in this study can be found in online repositories. The names of the repository/repositories and accession number(s) can be found below: https://www.ebi.ac.uk/arrayexpress/, E-MTAB-4591; https://www.ebi.ac.uk/arrayexpress/, E-MTAB-9044; and https://www.ebi.ac.uk/arrayexpress/, E-MTAB-9807.

## Ethics Statement

The studies involving human participants were reviewed and approved by the Medische Ethische Toetsings Commissie (METC), Erasmus MC. The patients/participants provided their written informed consent to participate in this study.

## Author Contributions

FR, MV, and LL designed the study. FR, HR, and LM acquired the data. FR, MV, and LL interpreted the data and wrote the draft of the manuscript. J-WP, GT, and JI were critical in obtaining material support. All authors provided intellectual content as well as critical revision.

## Conflict of Interest

The authors declare that the research was conducted in the absence of any commercial or financial relationships that could be construed as a potential conflict of interest.
