# Supplementary material for: Human Bile Contains Cholangiocyte Organoid-Initiating Cells Which Expand as Functional Cholangiocytes in Non-canonical Wnt Stimulating Conditions
Source: Front Cell Dev Biol. 2021 Feb 9;8:630492. doi: 10.3389/fcell.2020.630492 (PMC7900156; doi:10.3389/fcell.2020.630492)
Supplement: Supplementary file 1 [file Data_Sheet_1.PDF]

Supplementary Data

Material and methods

Generation and culture of canonical and non-canonical Wnt stimulated cholangiocyte organoids from bile and tissue

For the initiation (of culture) of bile cholangiocyte organoids in canonical-Wnt stimulating conditions (cBCOs, n=6), we used a protocol similar to the one previously published by Soroka *et al.*[1]. In short, organoids were initiated from a maximum of three mL of bile collected *ex vivo* and from one millilitre of bile collected *in vivo*. Bile was washed twice for five minutes with cold Advanced (Adv)DMEM/F12 (supplemented with 1% penicillin/streptomycin, Life Technologies; 1% Hepes 1 M, Fisher Scientific; 1% Ultra-glutamine 200mM, Fisher Scientific and 0.2% Primocin, Invivogen) at 453g and suspended in 8 mL of cold AdvDMEM/F12. Subsequently, the supernatant was removed and the pellet was suspended in 3 mL of AdvDMEM/F12 and passed through a 100 µm cell strainer to remove debris. After a third wash, the pellet was collected and plated out in a hydrogel (Matrigel, Corning, or diluted Basement Membrame Extract, BME, Cultrex). Medium was added according to the previously described protocol.[2,3] The first three days the medium was supplemented with 1% anti-anti (Gibco) to prevent bacterial- or fungal infections.

Tissue-derived intrahepatic cholangiocyte organoids in canonical-Wnt stimulating conditions (cICOs, n=3) and extrahepatic cholangiocyte organoids in canonical-Wnt stimulating conditions (cECOs, n=3) were initiated and cultured as previously published.[2-4] In short, liver or extrahepatic bile duct biopsies (0.5-1.0 cm) were digested using a collagenase solution in (2.5 mg/ml collagenase A1, Roche) EBBS (Hyclone, Thermoscientific) for 30 min at 37 ˚C. Digestion was stopped by adding cold supplement AdvDMEM/F12 filtered through a 70 µm cell strainer. The cell suspension was spun down for 5 minutes, 4˚C at 453*g*. Pellet was harvested in a hydrogel and specific medium was added according to protocol.[2,3] Medium was changed twice a week and organoids were passaged in a 1:2 to 1:8 ratio according to growth. Cultures were negative for mycoplasma contamination (data not shown).

The ncECO cultures (n=6) were initiated and maintained as previously published.[5] In short, cells were scraped from the inside of the gallbladders or from the inside of an extrahepatic bile duct (n=1) and collected in cold William's-E Medium (WE). The cells were centrifuged at 453*g* for 5 minutes at 4˚C, subsequently supernatant was removed, and the cell pellet was washed twice with excess cold WE. Next the pallet was collected and plated out in either matrigel or 70% BME diluted with WE and medium was added according to the standard protocol.[5]

Additionally, to investigate the influence of culture conditions on BCOs. Bile cholangiocyte organoids were either created from the same bile samples (n=3) either under cBCO conditions or ncBCO conditions. After three passages a switch from cBCO towards ncBCO conditions and from ncBCO conditions towards cBCO conditions was performed. Two passages later RNA was harvested from these 4 conditions per donor sample and analysed by qRT-PCR.

Using Chamber Measurement conditions

The temperature of the chambers was kept 37 °C by warm water bath circulation. Each chamber consisted of 3 mL modified Meyler solution (128 mmol/liter NaCl, 4.7 mmol/liter KCl, 1.3 mmol/liter $CaCl_2$, 1.0 mmol/liter $MgCl_2$, 0.3 mmol/liter Na_2HPO_4, 0.4 mmol/liter NaH_2PO_4, 20 mmol/liter $NaHCO_3$, 10 mmol/liter HEPES, supplemented with glucose (10 mmol/liter) in 95% O_2, 5% CO_2 at pH 7.3). Current was clamped and every second current was recorded. Two voltage spikes (5 millivoltage) were given, to measure resistance along the epithelial layers (*V=I*R; TEER*). CFTR-dependent anion secretion was activated by adding 20 µM Secretin (Sigma) basolateral or 10 µM Forskolin (Sigma) to

both sides of the cells and inhibited by addition of GlyH-101 (20 µM, apical side, Calbiochem) or 100 µM Somatostatin (Sigma) basolateral. Calcium (Ca^{2+}) depended chloride (Cl^-) channels (CaCC) were stimulated by UTP (50 µM, apical side, Sigma) and inhibited by T16Ainh-A01 (50 µM, apical side, Tocris).

Tables

Table S1. Donor and culture characteristics of organoids included.

Age patient (years)	Gender	Cells Source	Donor Type or Indication ERCP/surgery	Culture type	Successful Culture	Characterized at Passage #	Gene-expression by qRT-PCR
13	M	GB, GBB	DBD	ncECO 1, ncBCO 1	+	5-9	+
26	M	GBB	DCD	ncBCO 7**, cBCO 7**	+	5	+
29	M	ERCP	Bile stones	ncBCO 14	+	ND	-
30	M	ERCP	PSC	ncBCO 8 cBCO 8	+	5	+
35	F	GBB, GB	DBD	ncBCO 5, ncECO 5	+	5	+
41	F	EHBD	PSC	ncECO 11	+	5	+
47	M	GBB	DCD	ncBCO 6**, cBCO 6**	+	5	+
50	M	GB	Bile stones	ncECO 12	+	5-8	+
51	F	ERCP	AS	ncBCO 15	+	ND	-
52	M	GBB, GB	DCD	ncBCO 4, ncECO 4	+	5-9	+
54	M	EHBD	DBD	cECO D*	+	5	-
54	F	ERCP	Papiladenoma	ncBCO 10	+	5-8	+
56	F	EHBD	DCD	cECO C*	+	5	-
57	M	Liver, GBB, GBB	DCD	cICO 2, cBCO 2, ncBCO 2	+	5<br>5<br>5	+ (only cBCO)
58	F	ERCP	AS	ncBCO 16	+	ND	-
59	M	GB, GBB, GBB, Liver	DBD	ncECO 3, cBCO 3, ncBCO 3 cICO 3	+	5-8<br>5<br>5<br>5	+
67	M	Liver	DCD	cICO 13	+	5	+
67	F	EHBD	Cryptogenic Cirrhosis	cECO F*	+	5	-
70	M	ERCP	Bile stones	ncBCO 17	+	ND	-
72	M	ERCP	Bile stones	ncBCO 18	+	ND	-
73	F	ERCP	AS	ncBCO 19	-	ND	-
75	F	GBB	DBD	ncBCO 9**, cBCO 9**	+	5	+

Abbreviations: AS: Anastomotic bile duct Stricture, ncBCO: Bile Cholangiocyte Organoid in non-canonical Wnt stimulating conditions, EHBD: Extrahepatic Bile Duct, cECOs: extrahepatic cholangiocyte organoids in canonical Wnt stimulating conditions, cICOs: intrahepatic cholangiocyte

organoids in canonical Wnt stimulating conditions, cBCO: Bile Cholangiocyte Organoids organoids in canonical Wnt stimulating conditions, DBD: Donation after Brainstem Death, DCD: Donation after Circulatory Death, ncECO: Extrahepatic Cholangiocyte Organoids in non-canonical Wnt stimulating conditions, ERCP: Endoscopic Retrograde Cholangiopancreaticography, F: Female, GB: Gallbladder, GBB: Gallbladder Bile, M: Male, PSC: Primary Sclerosing Cholangitis, ND: Not Determined. *These organoid cultures were only analysed for Micro Array analysis. **Used for switching experiment (ncBCOs, are only used for the switching experiment).

Table S2. List of genes and primers used.

Gene		Primer sequence (5’ à 3’)
KRT7	F	GGGGACGACCTCCGGAATAC
	R	CTTGGCACGCTGGTTCTTGA
KRT19	F	GCACTACAGCCACTACTACACGA
	R	CTCATGCGCAGAGCCTGTT
HNF1β	F	TCACAGATACCAGCAGCATCAGT
	R	GGGCATCACCAGGCTTGTA
HPRT	F	GCTATAAATTCTTTGCTGACCTGCG
	R	CTTCGTGGGGTCCTTTTCACC
ALB	F	CTGCCTGCCTGTTGCCAAAGC
	R	GGCAAGGTCCGCCCTGTCATC
GAPDH	F	CTTTTGCGTCGCCAGCCGAG
	R	CCAGGCGCCCAATACGACCA
HNF4α	F	GTACTCCTGCAGATTTAGCC
	R	CTGTCCTCATAGCTTGACCT
AQP1	F	GGCCAGCGAGTTCAAGAAGAA
	R	TCACACCATCAGCCAGGTCAT
NKCC1	F	ACCAAGGATGTGGTAGTAAGTGTGG
	R	GGATTCTTTTTTCAACAGTGGTTGA
GGT	F	TGGTGGACATCATAGGTGGGGA
	R	ATGACGGCAGCACCTCACTT
ASBT	F	GGTGGCCTTTGACATCCTCCC
	R	GCATCATTCCGAGGGCAAGC
SOX9	F	ACCAGTACCCGCACTTGCAC
	R	GCGCCTTGAAGATGGCGTTG
EpCAM	F	GACTTTTGCCGCAGCTCAGGA
	R	AGCAGTTTACGGCCAGCTTGT
CFTR	F	TGGCGGTCACTCGGCAATTT
	R	TCCAGCAACCGCCAACAACT
LGR5	F	GTCAGCTGCTCCCGAATCCC
	R	TGAAACAGCTTGGGGGCACA
Cyp3A4	F	AGCAAAGAGCAACACAGAGCTGAA
	R	CAGAGGTGTGGGCCCTGGAAT
LOLX4	F	CTGGGCCGGACTGACTTTCGT
	R	CTTGTGCCCCTCAGCCACCTT
MMP1	F	GGCCCACAAACCCCAAAAGCG
	R	CGGGTAGAAGGGATTTGTGCGC
AGR2	F	TCCTAGCCGCCGACTCACACA
	R	GGTTTGACTGTGGTATCTCTGGCCA
CTSE	F	ATTGGCTCCCCACCACAGAA
	R	CTGGACTGGGAAGGCTGGAA

Gene		Primer sequence (5’à 3’)
IGFBP3	F	CAAGGGGAAGGAGGACGTGCA
	R	GCTGCTGGTCATGTCCTTGGC
KLK7	F	ACCACCTGTACTGTCTCCGGCT
	R	AACCTTCGTGCAGTCCTGGGG
IGFBP1	F	AGGCACAGGAGACATCAGGAGA
	R	CACCAGCAGAGTCCCGCCTC
SLC2a3	F	ATTGCTCTTCCCCTCCGCTGC
	R	CCTCAAAAGTCCTGCCACGGGT
TFF1	F	ACAAGCTGCTGTACACGGACA
	R	AAGTTTCCAGGGCCGGGCAAT
TFF2	F	TCTGTCCTGCCTCCCTGATCCA
	R	CTCTGGCACGTGAATCCCGGT
GC	F	ATGGCCAAAGAGCTGCCTGA
	R	TGGGCAGCTGGCATGAAGTA
A1AT	F	TGAGGAGAGCAGGAAAGGACA
	R	CTCAGCCAGGGAGACAGG
ALDH1A	F	TGAAGTCATCGGTCGGCTTGA
	R	ACCCCTACGATGACACTTGTGCC
LYZ	F	GGCAAAACCCCAGGAGCAGTT
	R	TGCCACCCATGCTCTAATGCCT
AGR2	F	TCCTAGCCGCCGACTCACACA
	R	GGTTTGACTGTGGTATCTCTGGCCA
S100A6	F	GGCAGGGAGGGTGACAAGCA
	R	CCTCCTGGTCCTTGTTCCGGT
TACSTD2	F	CGAGCTTGTAGGTACCCGGCG
	R	TGCGCCGAGGAATCAGGAAGC
LCN2	F	ACCACATCGTCTTCCCTGTCCC
	R	TGGCACTGTGGGGAGGGTCTC
KRT18	F	TCCAAAAGACCACCACCCGCC
	R	TGCTCCCCAAAGTGTACCCTGC
CES1	F	GCCCGGAACCACAGAGATGCT
	R	AGCTCATCCCCGTGGTCTCCT
CD133	F	CCTGGGGCTGCTGTTTATTA
	R	ATCACCAACAGGGAGATTGC
LGR5	F	GTCAGCTGCTCCCGAATCCC
	R	TGAAACAGCTTGGGGGCACA

Table S3. Overview of cholangiocyte organoids cultured in this manuscript.

Name	Abbreviation	Source	Culturing conditions	Protocol/ reference
Bile Cholangiocyte Organoid in canonical Wnt stimulating conditions	cBCO	Bile	Canonical Wnt	Soroka *et al.*[1]
Intrahepatic Cholangiocyte Organoid in canonical Wnt stimulating conditions	cICOs	Intrahepatic cholangiocytes obtained via a liver biopsy	Canonical Wnt	Huch *et al.*[2]; Broutier *et al.*[3]
Extrahepatic Cholangiocyte Organoid in canonical Wnt stimulating conditions	cECOs	Extrahepatic cholangiocytes obtained from an extrahepatic bile duct biopsy	Canonical Wnt	Rimland *et al.*[4]
Extrahepatic Cholangiocyte Organoid in non-canonical Wnt stimulating conditions	ncECO	Extrahepatic cholangiocytes	Non-canonical Wnt	Sampaziotis *et al.*[5]; Tysoe *et al.*[6]
Bile Cholangiocyte Organoid in non-canonical Wnt stimulating conditions	ncBCO	Bile	Non-canonical Wnt	This protocol

Figures

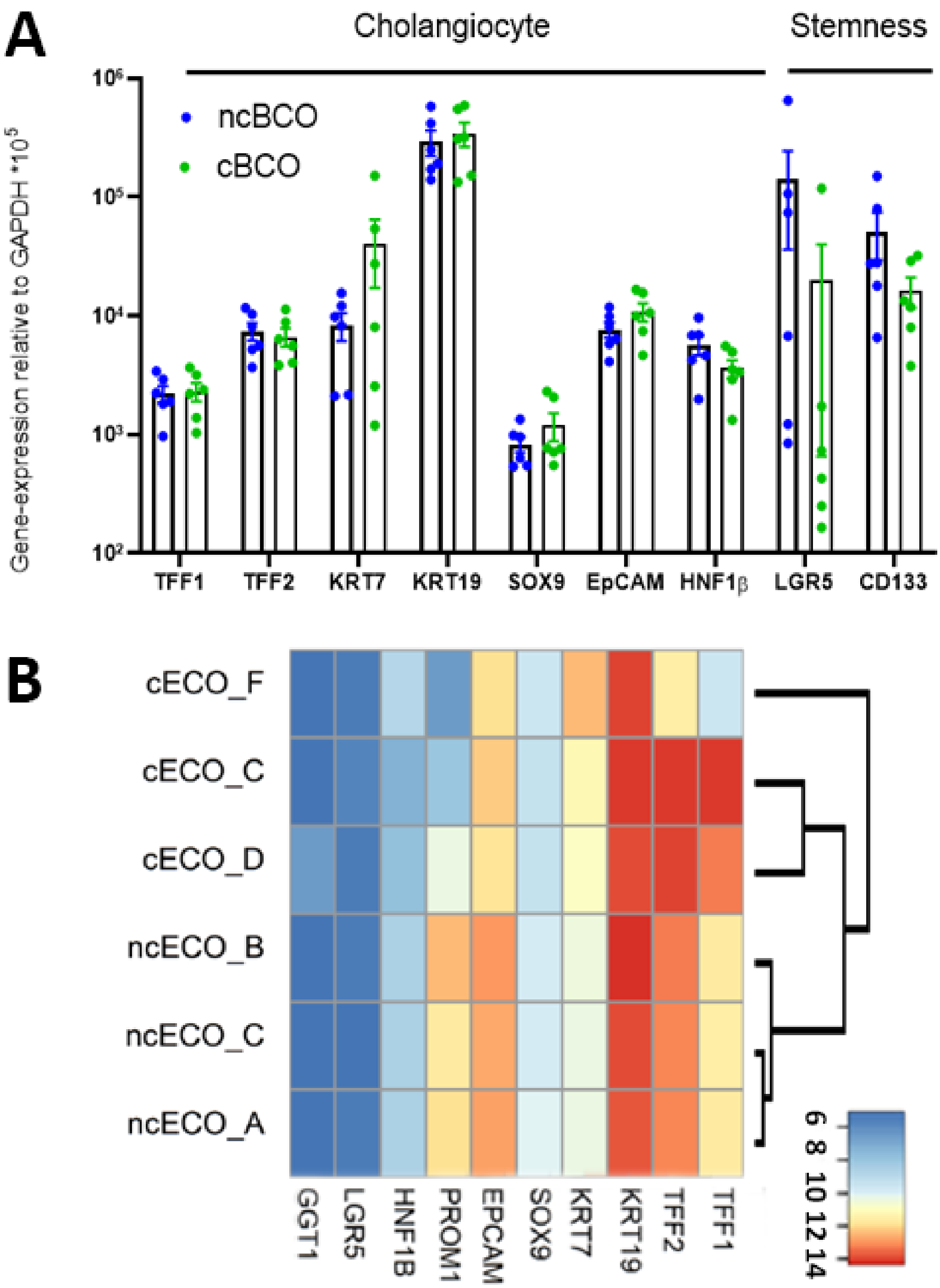

Supplementary Figure 1. Classical cholangiocyte markers are similar expressed between non-canonical Wnt and canonical-Wnt stimulating conditions.

(a) qRT-PCR showing comparison between ncBCOs (n=6) and cBCOs (n=6) for different cholangiocyte- and stemness/Wnt-target related genes, relative to the housekeeping gene *GAPDH*, error bars are presented with standard error of the mean. **(b)** Gene-expression as analysed by Micro Array for ncECOs (n=3) and cECOs (n=3) for the similar genes as displayed in panel **a**. Color key represents the log2 transformed signal intensities after variance stabilizing normalization.

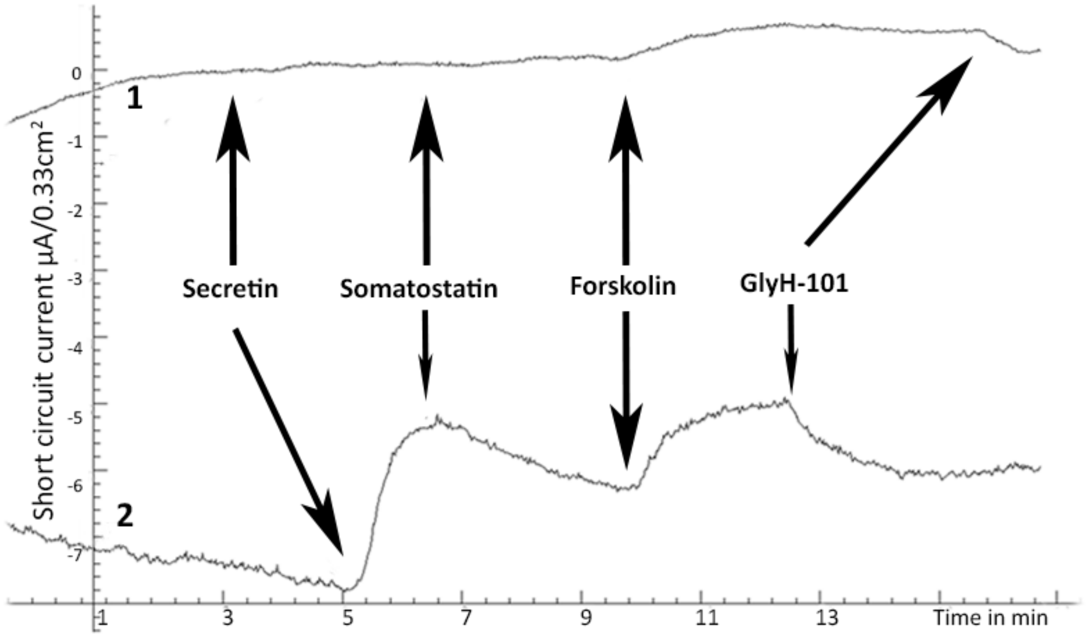

Supplementary Figure 2. Cholangiocyte related and ion-channel and basolateral receptor activity in ncECOs and cICOs.

Representative ion-channel functionality of 2D-grown tissue derived canonical-Wnt stimulated organoids (cICOs, line 1) and tissue derived non-canonical Wnt stimulated organoids (ncECOs, line 2) in an Ussing-chamber. Stimulation with cAMP-activator(forskolin), resulted in an increase in short circuit current, however secretin stimulation (to the basolateral side) only gave a response in the ncECOs. In similar fashion, somatostatin (basolateral addition) only give a response in ncECOs and not in cICOs, while Cystic Fibrosis Transmembrane conductance Regulator (CFTR)-inhibitor, GlyH-101 (luminal addition), resulted in an inhibition of the channel in both organoid-types. Indicating the presence of functional CFTR channels in both organoids, but only somatostatin and secretin-receptors are functional in ncECOs. Moreover, the CFTR responses in ncECOs seems more pronounced compared to cICOs.

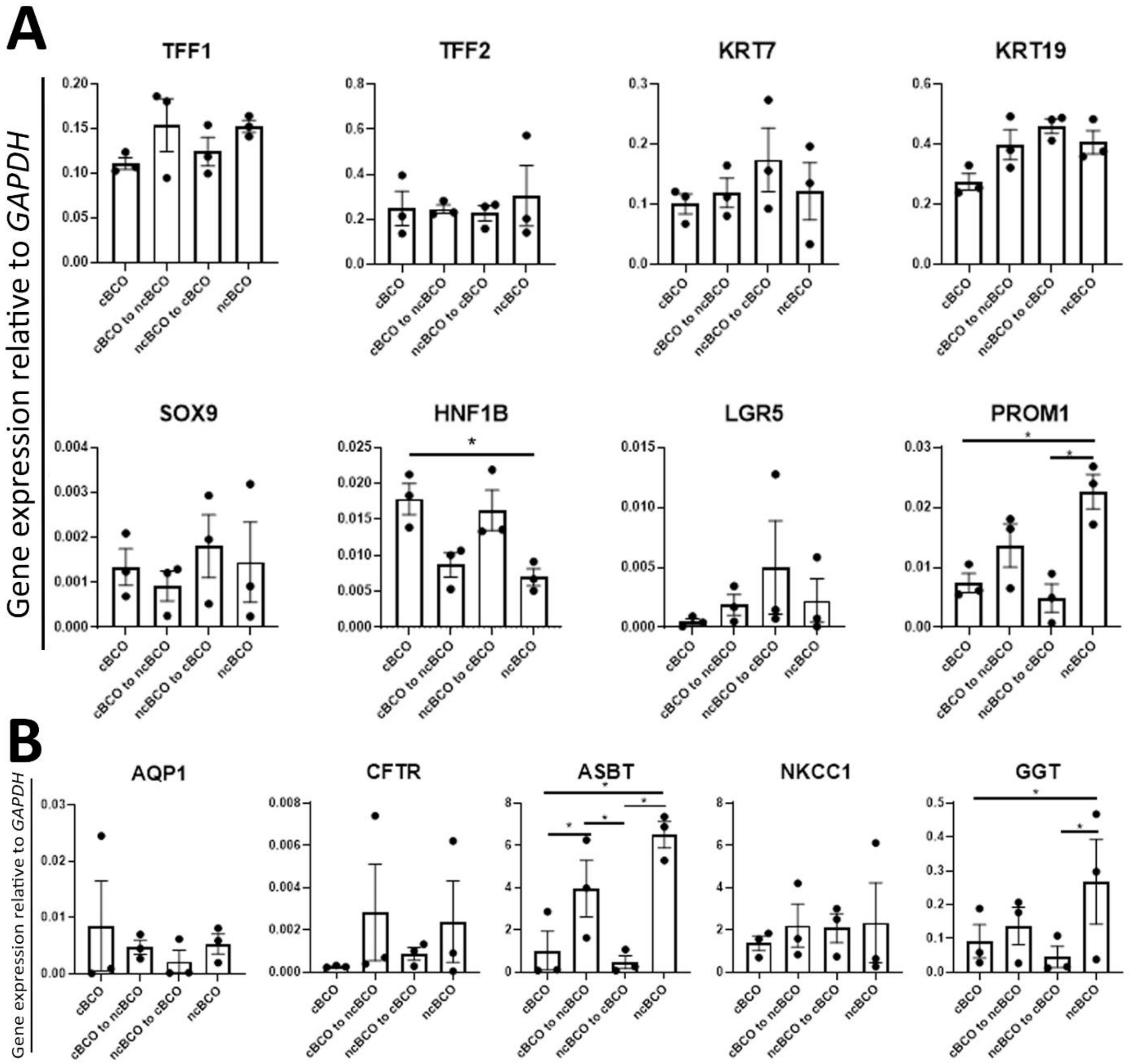

Supplementary Figure 3. Gene-expression profiles of bile cholangiocyte organoids under different culture conditions.

(a) Gene-expression profiles as analysed by qRT-PCR for BCOs started up in either canonical Wnt (cBCOs) or non-canonical Wnt culture (ncBCOs) conditions for the classical cholangiocyte and stem cell/Wnt-target genes. After three passages pallet was split in half and organoids were either continued in their original conditions or switch to the other (n=3, all conditions). Error bars indicate standard error of the mean. **(b)** Gene-expression profiles of the cholangiocyte markers related to function as analysed by qRT-PCR for the same samples as displayed in panel a. Error bars indicate standard error of the mean. *is considered a significant difference (p<0.05).

Supplementary references

1. Soroka CJ, Assis DN, Alrabadi LS, *et al.* Bile-Derived Organoids From Patients With Primary Sclerosing Cholangitis Recapitulate Their Inflammatory Immune Profile. Hepatology. 2019 Sep;70(3):871-882.
2. Huch M, Gehart H, van Boxtel R, *et al.* Long-Term Culture of Genome-Stable Bipotent Stem Cells from Adult Human Liver. Cell. 2015 Jan 15; 160(1-2): 299–312.
3. Broutier L, Andersson-Rolf A, Hindley CJ, *et al.* Culture and establishment of self-renewing human and mouse adult liver and pancreas 3D organoids and their genetic manipulation. Nat Protoc. 2016 Sep;11(9):1724-43.
4. Rimland CA, Tilson SG, Morell CM, *et al.* Regional differences in human biliary tissues and corresponding in vitro derived organoids. Epub ahead of print 29 March 2020.
5. Sampaziotis F, Justin AW, Tysoe OC, *et al.* Reconstruction of the mouse extrahepatic biliary tree using primary human extrahepatic cholangiocyte organoids. Nat Med. 2017 Aug;23(8):954-963.
6. Tysoe OC, Justin AW, Brevini T, *et al.* Isolation and propagation of primary human cholangiocyte organoids for the generation of bioengineered biliary tissue. Nat Protoc. 2019 Jun;14(6):1884-1925.
